# Neurite Branching Regulated by Neuronal Cell Surface Molecules in *Caenorhabditis elegans*

**DOI:** 10.3389/fnana.2020.00059

**Published:** 2020-08-21

**Authors:** HoYong Jin, Byunghyuk Kim

**Affiliations:** Department of Life Science, Dongguk University-Seoul, Goyang, South Korea

**Keywords:** neurite branching, neuronal cell surface molecule, *C. elegans*, receptor complex, actin dynamics, neural circuit formation

## Abstract

The high synaptic density in the nervous system results from the ability of neurites to branch. Neuronal cell surface molecules play central roles during neurite branch formation. The underlying mechanisms of surface molecule activity have often been elucidated using invertebrates with simple nervous systems. Here, we review recent advances in understanding the molecular mechanisms of neurite branching in the nematode *Caenorhabditis elegans*. We discuss how cell surface receptor complexes link to and modulate actin dynamics to regulate dendritic and axonal branch formation. The mechanisms of neurite branching are often coupled with other neural circuit developmental processes, such as synapse formation and axon guidance, *via* the same cell-cell surface molecular interactions. We also cover ectopic and sex-specific neurite branching in *C. elegans* in an attempt to illustrate the importance of these studies in contributing to our understanding of conserved cell surface molecule regulation of neurite branch formation.

## Introduction

An extensive neurite branching morphology is a fundamental aspect of neuronal structure. Each axon and dendrite contain numerous neurite branches that enhance neural circuit complexity by allowing for interaction with a large number of target neurons and non-neuronal cells. For example, a single neuron can synapse onto multiple target neurons due to the extensive branching of the axonal shaft. Dendrites have an extremely complex branching thereby producing a large dendritic field to receive synaptic or sensory inputs. These neurite branch networks allow for the formation of highly complex neural circuits that integrate and process information, thereby coordinating specific nervous system functions. Growing evidence suggests that dysregulation of neurite branching could underlie various neurological and neurodevelopmental disorders such as autism, schizophrenia, and Down syndrome (Kulkarni and Firestein, 2012; Copf, 2016).

Numerous previous studies have identified molecules that regulate neurite morphogenesis, including transcription factors, cell surface molecules, and regulators of actin and microtubule dynamics (Jan and Jan, 2010; Kalil and Dent, 2014). Of these molecules, cell surface proteins have been shown to modulate the precision of neural circuitry wiring *via* extracellular interactions (De Wit and Ghosh, 2016). Most neural cell surface molecules are evolutionarily conserved and play a critical role in neural circuit formation (Kim, 2019). Moreover, certain cell surface molecules can control various steps of circuit assembly (Kim, 2019). Evidence shows that the localization of neural cell surface receptors and their respective extracellular ligands is highly correlated with branch formation. Furthermore, these interactions are shown to control branch formation by stimulating or inhibiting nascent branch outgrowth. Subsequently, branch stabilization and outgrowth require intracellular reorganization of actin and microtubules, in which the actin cytoskeleton plays a role in an initial step of branch formation (Jan and Jan, 2010; Kalil and Dent, 2014). While the exact details of how extrinsic cues are transduced into intracellular signals through cell surface receptors to control actin dynamics during neurite branching remain poorly understood, recent findings in *Caenorhabditis elegans* begin to provide some insight.

Several features of the nematode *C. elegans* nervous system make it a powerful model for studying molecular mechanisms of neurite branching. First, because most *C. elegans* neurons have simple, unbranched morphologies, the few neurons with neurite branches can be observed with great specificity (White et al., 1986). Branching patterns of these neurons are highly stereotyped throughout development (Altun and Hall, 2011). Second, the entire nervous system structure and neural connectivity map (connectome) have been described in great detail in both sexes (White et al., 1986; Jarrell et al., 2012; Cook et al., 2019). This provides information about specific neural circuits and how synaptic connectivity is associated with branching morphologies. Third, the *C. elegans* genome contains about a hundred genes encoding neuronal cell surface proteins with extracellular interaction domains (Hobert, 2013). The majority of these genes are evolutionarily conserved and expected to function for cell surface recognition (Hobert, 2013). Fourth, *C. elegans* genetics are simple, and diverse genetic screening methods are available, facilitating the rapid identification of novel factors that act in specific genetic pathways (Jorgensen and Mango, 2002).

In this review, we focus on *C. elegans* neurobiology to highlight recent advances in the understanding of the molecular mechanisms that control neurite branching. Specifically, we discuss the regulation of actin dynamics by cell surface receptor complexes during axonal and dendritic branch formation. Furthermore, in an attempt to illustrate the importance of conserved cell surface molecule regulation of neurite branch formation, we discuss two specific types of neurite branching: ectopic branching and sex-specific branching.

### Dendritic Branching: A Multi-protein Ligand-Receptor Complex That Regulates Dendritic Arborization

**Figure 1:**
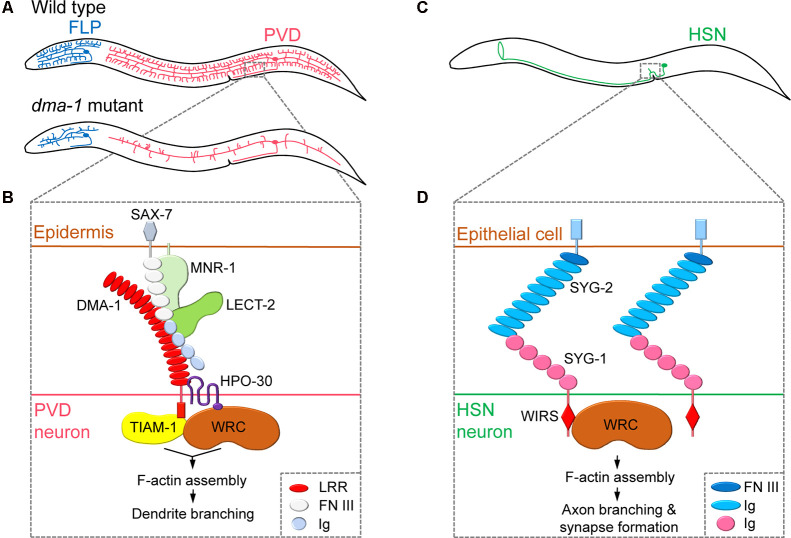
Regulation of neurite branching by neuronal cell surface molecules in dendrites and axons.** (A)** The position of the cell body and branching pattern of PVD (red) and FLP (blue). **(B)** The receptor-ligand complex consisting of DMA-1, SAX-7, MNR-1, LECT-2, and HPO-30 interacts with TIAM-1 and WAVE regulatory complex (WRC) to induce F-actin assembly, thereby promoting dendritic branching. Adapted from Zou et al. (2018). **(C)** The position of the cell body and branching pattern of HSN (green). **(D)** The interaction of SYG-1 and SYG-2 and the direct binding of the WRC interacting receptor sequence (WIRS) motif of SYG-1 with WRC mediates localized F-actin assembly, which promotes axonal branching and synapse formation. Adapted from Chia et al. (2014) and Özkan et al. (2014). LRR, leucine-rich repeat; FN III, fibronectin type-III; Ig, immunoglobulin.

Neurites of many *C. elegans* neurons both receive synaptic inputs and outputs, but some neuron processes have only sensory functions (dendrites) or synaptic output functions (axon; Altun and Hall, 2011). A well-characterized *C. elegans* neuron for neurite branching is a somatosensory neuron, termed PVD. The *C. elegans* nervous system contains two PVD neurons located on the lateral sides (both left and right) of the posterior section of the body. These neurons extend elaborate dendritic branches throughout the body excluding the head (Figure 1A). The *C. elegans* head is covered by a PVD-like branched neuron, termed FLP. Together, the PVD and FLP neurons comprise a sensory network of the entire body that responds to harsh mechanical stimuli (Way and Chalfie, 1989). PVD and FLP neurons show some differences in function and morphology: PVD neurons sense hot and cold temperature, high osmolarity and play a role in proprioception, whereas FLP neurons sense noxious high temperatures and humidity (Chatzigeorgiou et al., 2010; Albeg et al., 2011; Mohammadi et al., 2013; Cohen et al., 2014; Tao et al., 2019). Also, PVD is morphologically unciliated while FLP is ciliated (Ward et al., 1975; Altun and Hall, 2011).

Studies on PVD dendritic branching mechanisms have revealed that the neuronal cell surface protein DMA-1 is a central component of a multi-protein ligand-receptor complex that regulates branching. Mutants lacking the *dma-1* gene display reduced PVD branching phenotypes (i.e., reduced number of multiple short branches that normally arise from the main branches), leading to defects in harsh touch response (Liu and Shen, 2011; Figure 1A). DMA-1/LRR-TM is a transmembrane protein that contains extracellular leucine-rich repeat (LRR) domains and acts as a cell-autonomous receptor during PVD dendritic branching (Liu and Shen, 2011). A subsequent forward genetic screen identified two adhesion-type cell surface molecules, SAX-7/L1CAM and MNR-1/Menorin, which are expressed in the skin, as ligands for the PVD receptor DMA-1 (Dong et al., 2013; Salzberg et al., 2013). Moreover, chemokine LECT-2/Chondromodulin II, another identified ligand secreted from muscles, forms a macromolecular complex together with SAX-7, MNR-1, and DMA-1 (Díaz-Balzac et al., 2016; Zou et al., 2016). Mutations in genes encoding SAX-7, MNR-1, and LECT-2 also result in reduced PVD dendritic arbors (Salzberg et al., 2013; Díaz-Balzac et al., 2016). As a result, a current model of the extracellular signals that govern PVD dendritic branching has been developed. This model suggests that to initiate neurite branching, PVD neurites expressing DMA-1 receive extracellular signals *via* interactions with SAX-7 and MNR-1 in the skin, and, subsequently, LECT-2 protein secreted from muscles strengthens the binding of DMA-1 to SAX-7 and MNR-1 (Figure 1B).

After a branch point is determined, remodeling of the actin cytoskeleton is required to promote nascent neurite outgrowth. Indeed, a large body of research has identified the Rho family of small GTPases as a key actin regulator during neurite branching (Jan and Jan, 2010; Kalil and Dent, 2014). Two recent *C. elegans* studies have focused on the interacting partners of DMA-1 to provide insights into the effects of extracellular signaling on actin dynamics in PVD neurons (Zou et al., 2018; Tang et al., 2019). The DMA-1intracellular domain was shown to directly interact with TIAM-1/GEF, a conserved guanine nucleotide exchange factor (GEF) that regulates the activity of the small GTPase Rac to promote F-actin assembly (Zou et al., 2018; Tang et al., 2019). Interestingly, TIAM-1 functions in PVD branching independently of its GEF activity, possibly by direct interaction with actin (Tang et al., 2019). DMA-1 also interacts with the dendritic cell surface protein HPO-30/Claudin, which recruits the actin nucleation promotion factor WAVE regulatory complex (WRC) to induce F-actin assembly (Zou et al., 2018). Thus, the interaction between DMA-1 and HPO-30 facilitates the recruitment of two F-actin assembly regulators, TIAM-1 and WRC, into proximity to synergistically promote dendritic branching (Figure 1B). While many of the major molecular players are identified, the exact details of the molecular mechanisms that govern the transduction of extracellular signals through the DMA-1 receptor complex to regulate intracellular actin dynamics remain unknown.

While many of the molecular mechanisms that control PVD neurite branching have been identified, it is unknown whether other highly branched *C. elegans* neurons are governed by similar mechanisms. However, in addition to PVD neurons, studies have shown that depletion of the DMA-1 receptor complex components and associated proteins, including DMA-1, MNR-1, LECT-2, HPO-30, TIAM-1, and ACT-4/Actin, causes reduced branching phenotypes in FLP neurons, although these neurons have distinct branching architectures during development (Liu and Shen, 2011; Salzberg et al., 2013; Díaz-Balzac et al., 2016; Androwski et al., 2019; Tang et al., 2019; Figure 1A). Moreover, a proprotein convertase KPC-1/Furin, originally identified as a negative regulator of the DMA-1 complex pathway, was shown to promote both PVD and FLP branching by adjusting dendritic DMA-1 levels (Salzberg et al., 2014; Dong et al., 2016). KPC-1 is also required for the dendritic arborization of IL2 neurons, specifically induced during dauer stages (Schroeder et al., 2013). Recently, it was shown that the DMA-1 complex components are also required for IL2 arborization during dauer stages (Androwski et al., 2019). Therefore, it is likely that different neurons utilize similar DMA-1 receptor complexes to modulate dendrite morphogenesis; however, individual ligand-receptor complex components may differ.

### Axonal Branching: Cell Surface Molecules That Functionally Integrate Neurite Branching to Other Neural Circuit Formation Processes

By forming multiple neurite branches, neurons can increase the number of synaptic connections made with multiple target cells. Cell surface molecules can link synapse formation to neurite branching, as newly formed synapses can induce the formation and subsequent stabilization of branches. This complex behavior has been well studied in axonal branching of *C. elegans* HSN neurons. The axon of the hermaphrodite-specific neuron HSN extends dorsal branches that synapse onto the ventral cord motor neurons VC4 and VC5 and the vulval muscles, thereby modulating egg-laying behavior (White et al., 1986; Shen and Bargmann, 2003; Figure 1C). The Shen group has shown that HSN axonal branching and synapse formation are closely linked and mediated by a cell surface interaction between two immunoglobulin superfamily (IgSF) proteins, SYG-1 and SYG-2 (Shen and Bargmann, 2003; Shen et al., 2004; Chia et al., 2014). During HSN synaptogenesis, SYG-1 is required for correct synaptic vesicle localization and recruited to nascent synaptic regions *via* interactions with SYG-2, a guidepost signal that is temporarily expressed in primary vulval epithelial cells (Shen and Bargmann, 2003; Shen et al., 2004). As a result, the cell surface interaction between SYG-1 and SYG-2 specifies synapse formation at the correct position of the HSN axon. SYG-1/SYG-2 interaction also plays an important role in the formation of axon branching. The cytoplasmic tail of the SYG-1 protein contains a WRC interacting receptor sequence (WIRS), which directly binds to the actin nucleation promotion factor WRC. This interaction between SYG-1 and WRC directs the local assembly of F-actin (Chia et al., 2014). Interestingly, WRC is required for both synapse formation and axonal branch formation in HSN neurons. This suggests that extracellular SYG-1/SYG-2 interaction mediates local F-actin assembly through cytoplasmic SYG-1/WRC binding to mark synaptic and axonal branch positions (Chia et al., 2014; Figure 1D).

While conserved Netrin signaling plays a critical role during axon guidance, numerous studies indicate that it functions in multiple other neurodevelopmental processes, including synapse formation, extrasynaptic neurosecretory terminal targeting, and axonal branching (Hedgecock et al., 1990; Colón-Ramos et al., 2007; Nelson and Colón-Ramos, 2013; Chen et al., 2017). In *C. elegans*, secreted UNC-6/Netrin promotes synapse formation of the interneuron AIY that expresses the Netrin receptor UNC-40/deleted in colorectal cancer (DCC), an IgSF cell surface protein (Colón-Ramos et al., 2007). During serotonergic neuron NSM maturation, depletion of UNC-6 or UNC-40 leads to the reduction of axon arbors with the extrasynaptic neurosecretory terminals (Nelson and Colón-Ramos, 2013). Additionally, Netrin signaling induced by the UNC-6/UNC-40 interaction was shown to regulate the axonal branch growth of a touch mechanosensory neuron PLM *via* the promotion of F-actin assembly (Chen et al., 2017). Perturbation of Netrin signaling results in shorter or no branches at normal branching sites of several other *C. elegans* neurons (Hao et al., 2010). Importantly, Netrin receptors also have a WIRS domain, which directly binds WRC, suggesting that Netrin signaling can regulate the actin cytoskeleton (Chen et al., 2014). Taken together, these data illustrate that Netrin signaling is involved in axonal branching and multiple other neural circuit formation processes; however, the mechanistic link between these steps is less understood.

Another cell surface molecule that possibly links axonal branching to other neural circuit developmental processes is BAM-2, a transmembrane protein similar to the synaptic adhesion protein Neurexin. BAM-2 has been shown to regulate VC4 and VC5 axonal branch termination near the vulva, as depletion of BAM-2 causes VC axon extension beyond normal termination sites (Colavita and Tessier-Lavigne, 2003). Although the study found a cell non-autonomous function of BAM-2 in vulval cells, BAM-2 receptors have not been identified in VC neurons. BAM-2 was recently shown to interact with another cell surface molecule, the cadherin family protein CASY-1/calsyntenin. Moreover, this interaction mediates axon fasciculation and synapse formation in a male sensory circuit (Kim and Emmons, 2017). Future studies should examine if the axonal branching of VC neurons is also dependent on the BAM-2/CASY-1 interaction.

### Ectopic Neurite Branching: A Useful Model System for Identification of Conserved Cell Surface Molecules That Regulate Neurite Branching

**Figure 2:**
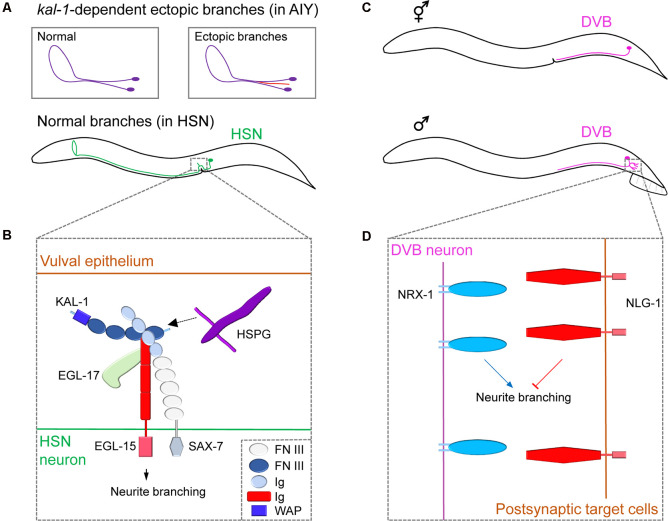
Neuronal cell surface mechanisms for neurite branching revealed by studying ectopic and sex-specific branches.** (A)** The morphology of the cell body and neurite branches of a normal AIY (violet) or ectopic AIY branch (red) generated by *Kal-1* overexpression (top); the normal branching pattern of HSN (green, bottom). **(B)** The complex of cell surface molecules KAL-1, EGL-15 and SAX-7 interact with HSPG and EGL-17, which promotes neurite branching in HSN. Adapted from Díaz-Balzac et al. (2015). **(C)** The position of the cell body and branching pattern of DVB (light violet) in the hermaphrodite (top) and male (bottom). **(D)** NRX-1 expressed in DVB promotes neurite branching, whereas NLG-1 expressed in postsynaptic target cells inhibits it. Adapted from Emmons (2018). FN III, fibronectin type-III; Ig, immunoglobulin; WAP, whey acidic protein.

Forced expression of neurite branching factors often results in ectopic branch formation. For example, in neurons with normally simple dendritic arbors, overexpression of the DMA-1 receptor complex components, such as DMA-1 or HPO-30, promotes ectopic branching (Liu and Shen, 2011; Smith et al., 2013). Similarly, forced expression of a branching factor KAL-1/Anosmin-1 in a normally-unbranched AIY neuron leads to the formation of extraneous branches (Bülow et al., 2002; Figure 2A). The subsequent forward genetic screening provided insights into the mechanisms of how conserved cell surface molecules act together to regulate neurite branching.

The human *KAL-1* gene encodes a secreted cell adhesion protein Anosmin-1 and, when mutated, is known to cause Kallmann syndrome, a genetic disease showing various behavioral and neurological defects (Hardelin, 2001). Loss-of-function or overexpressing mutants for the *C. elegans* *KAL-1* homolog showed extra-branching in several types of neurons (Rugarli et al., 2002). To investigate KAL-1 in *C. elegans*, the Bülow group conducted a genetic modifier screen using KAL-1-induced AIY branching as a model system. They discovered that ectopic branch formation is dependent on heparan sulfate proteoglycans (HSPGs) and their modifying enzymes (Bülow et al., 2002; Díaz-Balzac et al., 2014). HSPGs are cell-surface and extracellular matrix proteins with glycan chains and function in diverse aspects of nervous system development in both vertebrates and invertebrates (Saied-Santiago and Bülow, 2018). Using a similar modifier screen combined with a candidate approach, Bülow’s group further identified an IgSF protein, SAX-7/L1CAM, and the fibroblast growth factor receptor EGL-15/FGFR as members of the conserved KAL-1 receptor complex (Díaz-Balzac et al., 2015). Under normal conditions, KAL-1 acts as an autocrine co-factor for SAX-7 and EGL-15, together with HSPGs and EGL-17/FGF, to regulate HSN axonal branching (Figure 2B). These studies outline the successful identification of multiple conserved cell surface proteins that regulates neurite branching in a defined molecular pathway. Further studies on ectopic branch factors will help provide a greater understanding of branching mechanisms.

### Sex-Specific Branching: Well-Known Synaptic Adhesion Molecules That Shapes Branching Pattern Only in One Sex

While *C. elegans* has sex-specific neurons (eight in hermaphrodites; 91 in males), it also possesses 294 sex-shared neurons, some of which show notable sex differences in neuronal structure, branching pattern, and synaptic connectivity (Cook et al., 2019). For example, DVB, a GABAergic motor neuron, shows extensive sexual dimorphism in neurite branch morphology and connectivity (Figure 2C). Hermaphrodites display no DVB branches. However, males develop DVB neurite branches in adulthood and establish new synaptic connections with other neurons and muscles to control spicule movement, which is a male copulatory structure (Hart and Hobert, 2018). Consistent with the sexually dimorphic structures, DVB neurons function differently between sexes. Specifically, DVB neurons promote spicule protraction during mating in males, whereas, in hermaphrodites, they control defecation behavior (LeBoeuf and Garcia, 2017). Interestingly, DVB neurite outgrowth was shown to be dependent on the mating experience of males which affects the activity of DVB postsynaptic target cells or environmental stress such as starvation or high temperature during sexual maturation, suggesting a neurite branching regulatory mechanism that involves a dynamic interaction between a DVB neuron and its targets (Hart and Hobert, 2018; Hart, 2019).

Using a candidate-based approach, Hart and Hobert found that a pair of synaptic adhesion molecules, NRX-1/Neurexin, and its partner NLG-1/Neuroligin, regulate DVB branching (Hart and Hobert, 2018). Neurexin/Neuroligin interaction is believed to mediate synaptic maturation by linking two synaptic partner cells (Südhof, 2017). In *C. elegans*, NLG-1 is expressed in the postsynaptic DVB target cells and suppresses neurite branch formation, while NRX-1 is expressed in DVB to promote neurite branching (Figure 2D). This antagonistic relationship between Neurexin and Neuroligin suggests that the Neurexin/Neuroligin interactions regulate neurite branching in a synaptically independent manner. The underlying details of how Neurexin/Neuroligin interaction can separately govern neurite branching and synapse formation is currently unknown.

There are multiple other notable examples of sex-specific modulation of neuronal structures including PDB neurons (male-specific neurite branching), DD06 neurons (male-specific neurite branching), and PHC neurons (male-specific axon extension; Cook et al., 2019). Sexual dimorphism in neurite structures appears to produce functional behavioral differences as evidenced by the modulation of male PHC neurons for male mating behavior (Serrano-Saiz et al., 2017). Apart from the structural change of neurites, sex-specific synaptic connectivity in *C. elegans* was shown to be controlled by developmental mechanisms (e.g., pruning) and regulated by conserved cell surface molecules (e.g., Netrin and its receptor; Oren-Suissa et al., 2016; Weinberg et al., 2018). Future research can focus on how these neurons utilize several cell surface molecules to achieve sex-specific neurite pattern formation.

## Concluding Remarks

Over the past decades, studies have identified numerous cell surface molecule interactions implicated in neural circuit formation processes including neurite branching. Most of these molecules possess conserved structural domains, such as LRR, Ig domains, and cadherin repeats, which mediate protein-protein interactions necessary during neuronal morphogenesis (De Wit and Ghosh, 2016). All of the cell surface molecules discussed in this review have mammalian homologs, suggesting that the molecular mechanisms for neurite branching may also be conserved. Indeed, it has been shown that Netrin-1 (a mammalian homolog of UNC-6) and Anosmin-1 (a mammalian homolog of KAL-1) have branch-promoting activity in mammalian CNS neurons (Soussi-Yanicostas et al., 2002; Dent et al., 2004). The signal transduction mechanisms of neural cell surface molecules highlighted here appear to be common in axon and dendrite branching, as they link extracellular signals to actin dynamics during neurite branching initiation; however, individual ligand-receptor pairs controlling branch formation may differ in axons and dendrites. Diverse cell surface receptors, not covered in this review, such as Protocadherins, Roundabouts (ROBOs), G-protein coupled receptors (GPCRs), and ion channels, also contain the WIRS motif that directly binds WRC, suggesting that these proteins are also able to regulate the actin cytoskeleton (Chen et al., 2014). Several actin regulators, such as Rac and RhoA, and their roles in neurite branching have been extensively studied in invertebrates (Jan and Jan, 2010). Also, a recent study using a new cell-surface proteomics technique in the *Drosophila* brain revealed several conserved, but previously unidentified, cell surface molecules that act as regulators of neural circuit formation (Li et al., 2020). Therefore, we expect that, by adopting new technical advances and/or by using model organisms with a simpler nervous system, future studies will continue to identify and examine how conserved cell surface molecules linked to actin regulators control neurite branching during neurodevelopment. These advances will aid us in understanding how neuronal cell surface molecules coordinate to govern neurite branching and other circuit assembly processes and thereby organize functional neural circuits.

## Author Contributions

HJ and BK developed the concept. Both have written and edited the text.

## Conflict of Interest

The authors declare that the research was conducted in the absence of any commercial or financial relationships that could be construed as a potential conflict of interest.
